# Global Analysis of Type Three Secretion System and Quorum Sensing Inhibition of *Pseudomonas savastanoi* by Polyphenols Extracts from Vegetable Residues

**DOI:** 10.1371/journal.pone.0163357

**Published:** 2016-09-26

**Authors:** Carola Biancalani, Matteo Cerboneschi, Francesco Tadini-Buoninsegni, Margherita Campo, Arianna Scardigli, Annalisa Romani, Stefania Tegli

**Affiliations:** 1 Department of AgriFood Production and Environmental Sciences (DISPAA), Molecular Plant Pathology Laboratory, University of Florence, Via della Lastruccia 10, 50019 Sesto Fiorentino (Florence), Italy; 2 Department of Chemistry "Ugo Schiff", BioElectroLab, University of Florence, Via della Lastruccia 3, 50019 Sesto Fiorentino (Florence), Italy; 3 Consortium I.N.S.T.M., Via G. Giusti 9, 50121 Florence, Italy; 4 Department of Statistics, Computer Science, Applications "G. Parenti" (DiSIA)-PHYTOLAB (Pharmaceutical, Cosmetic, Food supplement Technology and Analysis), University of Florence, Via Ugo Schiff 6, 50019 Sesto Fiorentino (Florence), Italy; Virginia Tech, UNITED STATES

## Abstract

Protection of plants against bacterial diseases still mainly relies on the use of chemical pesticides, which in Europe correspond essentially to copper-based compounds. However, recently plant diseases control is oriented towards a rational use of molecules and extracts, generally with natural origin, with lower intrinsic toxicity and a reduced negative environmental impact. In this work, polyphenolic extracts from vegetable no food/feed residues of typical Mediterranean crops, as *Olea europaea*, *Cynara scolymus*, and *Vitis vinifera* were obtained and their inhibitory activity on the Type Three Secretion System (TTSS) and the Quorum Sensing (QS) of the Gram-negative phytopathogenic bacterium *Pseudomonas savastanoi* pv. *nerii* strain *Psn23* was assessed. Extract from green tea (*Camellia sinensis*) was used as a positive control. Collectively, the data obtained through *gfp*-promoter fusion system and real-time PCR show that all the polyphenolic extracts here studied have a high inhibitory activity on both the TTSS and QS of *Psn23*, without any depressing effect on bacterial viability. Extracts from green tea and grape seeds were shown to be the most active. Such activity was confirmed *in planta* by a strong reduction in the ability of *Psn23* to develop hyperplastic galls on explants from adult oleander plants, as well as to elicit hypersensitive response on tobacco. By using a newly developed Congo red assay and an ELISA test, we demonstrated that the TTSS-targeted activity of these polyphenolic extracts also affects the TTSS pilus assembly. In consideration of the potential application of polyphenolic extracts in plant protection, the absence of any toxicity of these polyphenolic compounds was also assessed. A widely and evolutionary conserved molecular target such as Ca^2+^-ATPase, essential for the survival of any living organism, was used for the toxicity assessment.

## Introduction

Plant pathogenic bacteria cause serious damages and heavy economic loss to the global agricultural production. Although they are less common than phytopathogenic fungi and viruses, bacterial control is a considerable challenge in agricultural practices. According to the first general principle of plant disease management, that is prevention, exclusion of the bacterial phytopathogens from their hosts is the primary control strategy. However, the application of very effective measures such as quarantine and eradication may have also a high economic impact [1]. Furthermore, the conventional control methods for phytopathogenic bacteria essentially still rely on the use of chemicals, mainly copper-based compounds and antibiotics. In Europe, antibiotics are not allowed for plant protection and copper is amongst the very few chemicals still authorized in organic agriculture [2]. Nevertheless, the use of copper was recently restricted as a result of its negative ecotoxicological impact, and for its effect on the increase of antibiotic-resistant bacteria into agroecosystems [3]. While promising alternatives to copper have been proposed against several phytopathogenic fungi, no sustainable options are yet available for phytopathogenic bacteria. Recently, many efforts were made to identify inhibitors which are able to interfere with virulence and pathogenicity bacterial systems and pathways; such efforts have mainly targeted the Quorum Sensing (QS) and the Type Three Secretion System (TTSS). In particular, QS allows bacteria to successfully communicate and thus to adapt their gene expression to biotic and abiotic stimuli [4]_._ The TTSS is a macromolecular complex essential for causing disease on susceptible plants. Through the TTSS, bacteria directly inject into the cytosol of host cells several pathogenicity and virulence effector proteins [5]_._

Salicylidene acylhydrazides (SAHs) are amongst the very few synthetic compounds that have been evaluated until now as an alternative to copper. In *Erwinia amylovora*, SAHs were demonstrated to target the TTSS gene expression, the secretion of effectors, and the needle assembly [6]. Similarly, several phenolic compounds were found to possess the ability to specifically alter TTSS gene expression, by acting as inhibitors, such as in *E*. *amylovora* [7] or as inducers, as for *Dickeya dadantii* [8]. Besides their role as bactericides, some plant phenol-based molecules, such as p-coumaric acid and salicylic acid, have been shown to interfere with the QS of the plant pathogens *Agrobacterium tumefaciens* and *Pectobacterium carotovorum* [9].

Increasing evidence suggests that several extracts from polyphenol rich plants, such as green tea, artichoke, olive tree and grapevine, have high antimicrobial activity [10–13]. For example, epigallocatechin gallate (EGCG) is the most abundant polyphenolic metabolite found in green tea. EGCG was shown to have high anti-virus and anti-bacterial activities against human pathogens. Furthermore, EGCG’s copper-like performance was verified against the causal agents of citrus canker and bacterial spot of tomato [10,14]. In this frame, great effort in current research is devoted to the development of anti-virulence compounds, both synthetic or from natural sources, instead of biocides. The control of human, animal, and plant bacterial pathogens would thus affect mechanisms that are not essential for bacterial viability, avoiding the risk of developing resistance, as it occurs with antibiotics and copper [15,16]. In the present study, polyphenolic extracts from *Olea europaea* and *Cynara scolymus* leaves were obtained by a green chemistry approach. Together with extracts from *Vitis vinifera* seeds and green tea (*Camellia sinensis* leaves), they were characterized in their polyphenolic composition, by HPLC/DAD (High-Performance Liquid Chromatography/Diode-Array Detection) and by HPLC/ESI-MS (High-Performance Liquid Chromatography/Electrospray Ionization Mass Spectrometry).

For the first time, these polyphenolic extracts were tested for their inhibitory activity on the TTSS and QS of the Gram-negative phytopathogenic bacterium *Pseudomonas savastanoi*, the causal agent of oleander and olive knot disease. The promoter activity of TTSS and QS on key genes was determined by using specific fusion constructs with the gene coding for the green fluorescent protein (GFP). In addition, inhibition of the TTSS expression and of other virulence and pathogenicity related genes was assessed by quantitative real-time PCR. The ecotoxicological profile of the investigated polyphenolic compounds was also evaluated, in view of their potential application in plant disease control.

## Materials and Methods

### Sources of the screened polyphenolic extracts

The extract from *O*. *europaea* L. (varieties Frantoio and Carboncella) (hereafter denoted as FO) was obtained using green leaves, collected at the Azienda Agricola Frantoio “Il Forbiciaio” (Piancastagnaio, Siena, Italy, 42°51′N 11°41′E) and Vitasafer srl (Montorio Romano, Rieti, Italy, 42°08′16″N 12°48′28″E). The artichoke leaves extract (FC) (cultivars Terom and Violetto) were obtained by Consorzio Carciofo Violetto Brindisino (Mesagne, Brindisi, Italy, 40° 33' 35,28'' N, 17° 48' 32,76'' E). The grape seeds dry extract (VN) was commercially available and purchased from SOCHIM International S.p.A. (Milano, Italy). The green tea leaves dry extract (TV) was also commercially available, under the name TEAVIGO^®^ (DSM Nutritional Products, Heerlen, Netherlands). At the moment, both extracts are commercialised for cosmetic and nutraceutical purposes.

### Solvents and reagents

All the solvents (HPLC grade) and formic acid (ACS reagent) used for the extraction process were purchased from Aldrich Chemical Company Inc. (Milwaukee, Wisconsin, USA). The standards for tyrosol, luteolin 7-*O*-glucoside, chlorogenic acid, gallic acid, (+) catechin and oleuropein were purchased from Extrasynthèse S.A. (Lyon, Nord-Genay, France). EGCG, epicathechin, cynarin, caffeic acid, hydroxytyrosol and p-coumaric acid were purchased from Sigma-Aldrich Co. (St. Louis, MO, USA). The HPLC-grade water was obtained via double-distillation and purification with a Labconco Water Pro PS polishing station (Labconco Corporation, Kansas City, USA).

### Extraction and fractioning of the polyphenolic extracts

The extraction for FO and FC was performed in a Rapid Extractor Timatic series (Tecnolab S.r.l., Perugia, Italy), using a solid-liquid extraction technology. The extraction was performed with water, in a stainless steel basket at a temperature of 60°C. The working cycle was fully automatic and switched between a dynamic phase, obtained with a set pressure (7–9 Bar), and a static phase necessary for transferring the substance into the extraction solvent. Forced percolation was generated during the stationary phase, which ensures a continuous flow of solvent to the interior of the plant matrix. This avoided over-saturation and the formation of preferential channels, thus ensuring total extraction of the active principles from the vegetal matrix.

### HPLC/DAD and HPLC/ESI-MS analysis

The HPLC/DAD analyses were performed with a HP 1100 liquid chromatograph equipped with a DAD detector (Agilent Technologies, Palo Alto, CA). In detail, the analytical column used for FO and VN was a LiChrosorb RP18 250×4.60 mm, 5μm (LichroCART, Merck Darmstadt, Germany) maintained at 26°C. The eluents were H_2_O adjusted to pH 3.2 by HCOOH (A), and CH_3_CN (B). For FO, a four-step linear solvent gradient was used starting from 100% A up to 100% B, for an 88-min period at a flow rate of 0.8 ml/min, as previously reported [17]_._ The VN extract was analyzed after solubilization in 70% EtOH pH 3.2 by HCOOH. A 7-step linear solvent gradient system, starting from 100% A up to 100% B was applied during a 117-min. period at a flow rate of 0.8 ml/min [18]. For the analysis of the FC and TV, a Luna C18 column 150×3.0 mm, 5μm (Phenomenex, Bologna, Italy) operating at 27°C was used. The eluents were H_2_O adjusted to pH 3.2 by HCOOH, and CH_3_CN. A three-step linear solvent gradient was performed starting from 100% H_2_O up to 100% CH_3_CN, with a flow rate of 0.6 ml/min for a 30-min. period [19]. The HPLC-MS analyses were performed using a HP 1100 liquid chromatograph, equipped with a DAD and a 1100 MS detectors. The interface was an HP 1100 MSD API-electrospray (Agilent Technologies, Santa Clara, United States). Mass spectrometer operating conditions were the following: gas temperature 350°C at a flow rate of 10.0 l/-min, nebulizer pressure 30 psi, quadrupole temperature 30°C and capillary voltage 3500 V. The mass spectrometer operated in positive and negative ionization mode at 80–120 eV.

### Qualitative and quantitative analysis of the polyphenolic extracts

The phenolic compounds that were present in these extracts were identified by using data from HPLC/DAD and HPLC/MS analyses, by comparing and combining their retention times, UV/Vis and mass spectra with those of several commercial standards. Each compound was quantified by HPLC/DAD, using a five-point regression curve built with the available standards. Calibration curves with r^2^ ≥0.9998 were considered. In all cases, the actual concentrations of derivatives were calculated after making corrections for changes in molecular weight. In particular, secoiridoid molecules for FO were calibrated at 280 nm, using oleuropein as a reference; elenolic acid derivatives at 240 nm using oleuropein; hydroxytyrosol, lignans and derivatives were calibrated as tyrosol at 280 nm; verbascoside and other hydroxycinnamic derivatives were calibrated at 330 nm using chlorogenic acid as a reference; flavonoids were calibrated with luteolin 7-*O*-glucoside at 350 nm. For VN, gallic acid was calibrated at 280 nm using gallic acid as reference; catechin, epicatechin and procyanidins were calibrated at 280 nm using (+) catechin as reference. For FC, chlorogenic acid, mono- and di-caffeoylquinic acids were calibrated at 330 nm with chlorogenic acid as a reference; cynarin was calibrated at 330 nm with the pure standard, and flavonoids at 350 nm with luteolin 7-*O*-glucoside. For TV, EGCG and epicatechin gallate (ECG) were calibrated at 280 nm using EGCG as a reference. The evaluation of the polyphenol content was carried out in triplicate. The results were recorded as mean values with the standard deviation.

For each extract, the concentrations (expressed as μmol/g of total polyphenols), as well as the dilutions in water to obtain solutions 100 μM in polyphenols (or any other appropriate concentration according to the tests performed), were calculated by summing the concentrations of the individual polyphenolic compounds here characterized, expressed in μmol/g, according to the HPLC/DAD data and based on the molecular weight of each compound.

### Bacterial strains, media and growth conditions

The bacterial strains used in this study are listed in Table 1. The bacteria were long-term stored at -80°C, in 40% (v/v) glycerol. Wild type *P*. *savastanoi* pv. *nerii* strain *Psn23* and its ∆*hrpA* mutant were routinely grown at 26°C, as liquid or solid cultures on King’s B medium (KB) [20], or on hrp-inducing minimal medium (MM) [21] to activate *in vitro* the expression of TTSS genes. *Escherichia coli* strains TOP10 and ER2925 were grown in Luria–Bertani (LB) liquid or agarose medium [22]. According to the experimental purposes and as required for plasmid maintenance, antibiotics were added to the growth medium at the following concentrations: streptomycin 10 μg/ml for *E*. *coli*, nitrofurantoin 50 μg/ml for *P*. *savastanoi*, and gentamicin 10 μg/ml in both bacterial species when transformed with the plasmids reported in Table 1. Any bacterial contamination was excluded by periodical monitoring using PCR-based assays specific for *P*. *savastanoi* [23].

**Table 1:** Bacterial strains, mutant and plasmids used in this study.

Strains/Plasmids	Relevant characteristics^§^	Reference/Source^˄^
**Strains**		
*E*. *coli*		
*E*. *coli* TOP10	F-, mcrA, Δ(mrr‐hsdRMS-crBC) Φ80 lacZΔM15 ΔlacX74 recA1 araD139 Δ(araleu)7697 galUgalKrpsL (StrR) endA1 nupG	Invitrogen, Carlsbad, USA
*E*. *coli* ER2925	ara-14 leuB6 fhuA31 lacY1 tsx78 glnV44 galK2 galT22 mcrA dcm-6 hisG4 rfbD1 R(zgb210::Tn10)TetS endA1 rpsL136 dam13::Tn9 xylA‐5mtl‐1 thi-1 mcrB1 hsdR2	NEB, Hertfordshire, UK
*P*. *savastanoi pv*. *nerii Psn23*	Wild type	LPVM collection
*∆hrpA*	*Psn23* in-frame deletion mutant for *hrpA*	[24]
**Plasmids**		
pBBR1-MCS5	broad host range replicating mobilisable vector, Gm^R^	[25]
pKEN *gfp*mut3	Ap^R^, mutated *gfp* (S65G, S72A)	[26]
pLPVM	Gm^R^, lacZ, mcs, *gfp*	This study
pLPVM_T3ApLPVM_QS	Gm^R^, lacZ, mcs, *hrpA* promoter+*gfp*Gm^R^, lacZ, mcs, *psnI* promoter+*gfp*	This studyThis study

^**˄**^LPVM Laboratorio di Patologia Vegetale Molecolare (University of Florence).

^**§**^Gm^R^, gentamicin resistance; Ap^R^, ampicillin resistance.

### Construction of plasmids

Extraction of genomic or plasmid DNA, PCR and general DNA manipulations, such as restriction digestion and ligation, were performed according to standard procedures and in accordance with the manufacturer’s instructions [27]. Cloning vector pBBR1-MCS5 was linearized with a double digestion using *KpnI* and *BamHI* enzymes. The primer pair GFP_BamHI_For / GFP_KpnI_Rev was used to amplify *gfp* gene, using the pKEN GFP mut3 plasmid as a template. Afterwards, the *gfp* amplicon was ligated into the linearized pBBR1-MCS5 in order to obtain pLPVM plasmid. A 102 bp fragment containing the promoter region for *hrpA*, and a 630 bp fragment containing the promoter for the *luxI* homologue in *Psn23* (hereafter named *psnI*), were amplified using the primer pair T3_XbaI_For / T3_BamHI_Rev and QS_XbaI_For / QS_BamHI_Rev, respectively (S1 Table), which were designed according to the *Psn23* sequences deposited in GeneBank (Accession Number FR717897.2 and FR717654). The amplified fragments were then cloned into the pLPVM plasmid, following their double digestion with *XbaI* and *BamHI*, to drive the expression of the promoter-less *gfp* gene, and to create the promoter-probe constructs pLPVM_T3A and pLPVM_QS. After their sequences were validated, these recombinant plasmids were transferred into *E*. *coli* ER2925, and then electroporated into *Psn23* with Gene PulserXCell™ (Bio-Rad Laboratories Inc., Hercules, CA, USA).

### Antibacterial activity test

The antibacterial activity of the polyphenolic extracts VN, TV, FO, and FC was evaluated *in vitro* by monitoring the bacterial growth as optical density at 600 nm (OD_600_), at different times during 24-h incubation with the polyphenolic extracts herein tested using the spectrophotometer Infinite^®^ M200PRO Quad4 Monochromators™-based (TECAN, Switzerland). Moreover, as references for these extracts, catechin, epicatechin, EGCG, oleuropein, caffeic acid, chlorogenic acid, cynarine, and luteolin 7-*O* glucoside, were tested as well. The *Psn23* cells were cultured in KB medium at 26°C overnight, and after two washes in sterile physiological solution (SPS, 0.85% NaCl in distilled water) the bacterial pellet was resuspended adjusting to a final OD_600_ = 0.5 in MM, supplemented or not with the polyphenolic extracts or with other standard molecules, at concentrations ranging from 1 to 100 μM. As control, the antibiotic kanamycin was also used (100 μg/ml).

### Hypersensitive Response assay

Hypersensitive Response (HR) assay was performed on *Nicotiana tabacum* (var. Burley White), grown at 24°C, with a relative humidity of 75% and a photoperiod of 16/8-h light/dark. The polyphenolic extracts VN, TV, FO, and FC were diluted up to 100 μM in sterile distilled water, and a 100 μl aliquot was then co-infiltrated with *Psn23* OD_600_ = 0.5, (approximately 0.5x10^8^ Colony Forming Unit/ml; CFU/ml), by using a needleless syringe into the abaxial mesophyll of fully expanded leaves of three tobacco plants [28]. The development of typical HR necrotic and chlorotic symptoms was monitored, and photographic records of the results were obtained at 24 and 48-h post-inoculation.

### Pathogenicity test

To test any variation in the ability of *Psn23* to cause the typical hyperplastic symptoms on oleander following the treatment with the polyphenolic extracts here studied, a plant model system was developed efficiently mimicking what occurring when using the whole host plant. Explants having a length of about 10 cm were collected from 2-year-old twigs of *Nerium oleander* plants (var. Hardy Red), washed three times under vacuum in sterile water supplemented with Tween^®^20 (SIGMA-Aldrich Co.), 1% sodium hypochlorite and 10% penconazole, and finally dried on sterile filter paper. These explant were then cut into 3–3.5 cm pieces by carefully removing their ends, weighted and placed on agar-H_2_O medium (7 g/l), amended with 10% penconazole, into sterile Magenta^TM^ GA-7 plant culture boxes (bioWORLD, Dublin, Ohio, USA). The upper end of each explant was then inoculated with 10 μl of a bacterial suspension, at a density of OD_600_ = 0.5, mixed or not with each polyphenolic extract (100 μM). The ∆*hrpA* mutant of *Psn23* was used as control. The inoculated oleander explants were then incubated in a growth chamber at 26°C, 75% relative humidity and a photoperiod of 16/8-h of light/dark. Symptoms were recorded at 21 days post-inoculation (dpi). At this time, the explants were weighed, and the weight increment recorded.

Nine replicates were used for each treatment, and three independent experiments were performed.

### GFP-based transcriptional screening

The *Psn23* bacterial cells carrying the promoter-probe plasmids pLPVM_T3A or pLPVM_QS (Table 1) were cultured overnight on KB medium at 26°C. Then their pellet was washed twice with SPS, and inoculated in MM (final OD_600_ = 0.5) supplemented with polyphenolic extracts VN, TV, FO, or FC, or their reference molecules, at concentrations ranging from 1 to 100 μM. Wild type *Psn23* carrying the empty vector was used as control. The experiments were carried out into 24 multiwell plates (BIOFIL^®^, Guangzhou, China) at different time during 24-h of incubation. The promoter activity of *hrpA* and *psnI* were then analyzed and quantitatively assessed, using the multimode microplate reader Infinite^®^ M200PRO Quad4 Monochromators™-based (TECAN), by simultaneously measuring the GFP intensity and the bacterial growth.

### Quantitative gene expression analysis

Starter liquid cultures of strain *Psn23* were grown overnight at 26°C on 20 ml KB, with continuous shaking at 100rpm. Cells were washed twice in SPS and used to inoculate MM medium, alone or supplemented with 100 μM of the polyphenolic extracts VN, TV, FO, or FC, to a final concentration OD_600_ = 0.5 (approximately 0.5x10^8^ CFU/ml). The bacterial cultures were then incubated for 24-h at 26°C. Total RNA was purified from bacteria during their stationary phase (OD_600_ = 1), using NucleoSpin^®^ RNA Plus (Macherey-Nagel GmbH and Co. KG, Düren, Germany). Residual genomic DNA was eliminated by a further treatment with NucleoSpin^®^ gDNA Removal Column (Macherey-Nagel GmbH and Co). The RNA quality was evaluated both spectrophotometrically, with NanoDrop™ ND-1000 (NanoDrop Technologies Inc., DE, USA), and visually by standard agarose gel electrophoresis [1% agarose (w/v) in TBE 1×][27]. About 1 μg of RNA for each treatment was reverse transcribed, using iScript™ Advanced cDNA Synthesis kit (Bio-Rad Laboratories Inc., Hercules, CA, USA), according to the manufacturer’s instructions. Diluted cDNA was analysed by real-time PCR, with SsoFast™ EvaGreen^®^Supermix (Bio-Rad Laboratories Inc.) and using the CFX96 cycler–real-time PCR Detection System and CFX-manager software v1.6 (Bio-Rad Laboratories Inc.). The primer pairs used are listed in S2 Table. The expression of each monitored gene was normalised with *16S rDNA*, as previously reported [29]. PCR conditions were 95°C for 10s and 60°C for 20s. The melting curves of the PCR products were acquired by a stepwise increase in temperature from 60 to 95°C, with a 0.5°C increase every 5s, at the end of each PCR run to check for aspecific amplifications. The specificity of each primer pairs was confirmed according to the single peak constantly produced in their melting curves, as shown in S2 Fig. To analyze the mRNA levels the comparative Livak (2^- ∆∆Ct^) method was used [30]. The fold induction of the mRNA of each target gene was determined form the threshold cycle values (Ct) of the housekeeping gene, and then for the fold expression of the wild type strain imposed as = 1, to obtain a relative expression data for each gene examined. The use of the 2^- ∆∆Ct^ method for relative quantification, a comparative technique in which a target gene is normalized to an endogenous control, requires the PCR efficiencies of target and control genes to be approximately equal. To verify this condition and to avoid significant measurement inaccuracies, ten-fold dilution series of *Psn23* genomic DNA (from 50 ng to 0.5 pg) were amplified to evaluate the amplification efficiency by comparison of the slope of the standard curves of target genes (*hrpA*, *hrpL*, *hrpRS*, *hrpV*, *lon*, *rpoN*, *pssR*, *pssI*) and the reference gene *16S rDNA*. The slope of the linear regression and the correlation coefficient for each curve are reported in S2 Table.

### Congo Red assay

*Psn23* cells were grown on MM liquid medium (OD_600_ = 0.2), supplemented or not with the polyphenolic extracts here examined (100 μM), and incubated at 18°C for 24-h with continuous shaking (100rpm). After 24-h incubation, the concentration of bacterial cultures was evaluated as OD_600_, and then the dye Congo red (SIGMA-Aldrich Co.) was added (10 μg/ml), followed by a further incubation at 18°C for 1-h, under shaking. Bacterial cells were removed by centrifugation (5000*g* for 10min.), and 1 ml supernatant for each sample was then aliquoted into 24 multiwell plates (BIOFIL^®^). The absorbance value at 490 nm (OD_490_) was recorded by spectrofluorimetry using Infinite^®^ M200PRO (TECAN). The Congo red binding is directly correlated both to the TTSS pilus assembly and to the bacterial concentration [31]. Therefore, for any treatment the value obtained was calculated as a percentage of the binding ability of the wild type *Psn23* and of its mutant *ΔhrpA*, according to the formula:

$$\frac{\left({\text{X}}_{\text{unk}}\text{-}{\text{X}}_{\Delta \text{hrpA}}\right)}{\left({\text{X}}_{\text{WT}}\text{-}{\text{X}}_{\Delta \text{hrpA}}\right)}\times 100$$

where X_WT_ and X_ΔhrpA_ are the ratio OD_490_/OD_600_ for *Psn23* and *ΔhrpA* respectively, untreated with any polyphenolic extract.

### ELISA assay

After an overnight growth at 26°C on KB medium, *Psn23* cells were washed twice, and inoculated in MM (OD_600_ = 0.5), supplemented or not with the polyphenolic extracts VN, TV, FO, or FC (100 μM), and grown at 18°C for 24-h under shaking. Cells were removed by centrifugation (8000*g* for 15min., at 4°C). The supernatant was then filtered through a 0.45 μm membrane (Filtropur S., Sarstedt, Nümbrecht, Germany) and 100 μl were then used to perform ELISA assay. Polyclonal primary antibodies against HrpA protein of *Psn23* were obtained from Primm srl (Milano-Italy), following immunization of two rabbits with recombinant protein HrpA. Secondary anti-rabbit horseradish peroxidase conjugate antibodies were used (Bethyl Laboratories Inc., Montgomery, TX, USA), according to manufacturer’s instructions. The standard curve was obtained with serial dilution of HrpA recombinant purified protein. The experiment was performed three times, with two replicates for each treatment.

### Current measurements on a solid supported membrane

The polyphenolic compounds here examined were investigated for their effects on Ca^2+^-ATPase, taken as a model of the ubiquitous molecular ion pumps P-type ATPases, known to be targets for many toxic compounds. Current measurements were carried out on sarcoplasmic reticulum (SR) vesicles containing Ca^2+^-ATPase adsorbed onto a hybrid alkanethiol/phospholipid bilayer anchored to a gold electrode (the so-called Solid Supported Membrane, SSM) [32]. SR vesicles were adsorbed on the SSM surface during an incubation time of 60min. Ca^2+^-ATPase was then activated by the rapid injection of a solution containing ATP. If at least one electrogenic step, *i*.*e*. a net charge movement within the protein, is involved in the relaxation process that follows protein activation, a current signal is recorded due to the capacitive coupling between vesicle membrane and SSM. It should be pointed out that the current amplitude is related to the number of adsorbed ATPase molecules that are activated after the ATP concentration jump, and the associated charge, which is obtained by numerical integration of the current signal, corresponds to the overall amount of Ca^2+^ ions translocated by the proteins following their activation [33]. In ATP concentration-jump experiments two buffered solutions were employed, the “non-activating” and the “activating” solution: the non-activating solution contained 100 mM KCl, 25 mM MOPS (pH 7.0), 1 mM MgCl_2_, 0.25 mM EGTA and 0.25 mM CaCl_2_ (10 μM free Ca^2+^); in addition, the activating solution contained 100 μM ATP. To investigate the effects of reference polyphenolic compounds on current signals generated by Ca^2+^-ATPase, the required concentration of each compound was added to both the non-activating and activating solutions. The ATP-induced current signal observed in the presence of the polyphenolic compound was compared to the control measurement obtained in the absence of the compound. To prevent Ca^2+^ accumulation into the vesicles, 1 μM calcium ionophore A23187 (calcimycin) was used. The concentration jump experiments were performed by the SURFE^2^R^One^ device (Nanion Technologies, Münich, Germany). The temperature was maintained at 22–23°C for all the experiments. To verify the reproducibility of the current signals on the same SSM, each single measurement was repeated six times, and then averaged to improve the signal to noise ratio. Standard deviations did not exceed 5%.

### Statistical analysis

All the experiments in this study were performed in triplicate and repeated three times, unless otherwise stated. The data were presented as the means ± standard deviation (SD) and subjected to one-way analysis of variance (ANOVA) using PAST software (Version 3.11, Øyvind Hammer, Natural History Museum, University of Oslo). When ANOVA indicated a significant difference (*P* < 0.05), a Tukey-Kramer post-test was performed.

## Results and Discussion

### Characterization of polyphenolic extracts from olive, artichoke leaves, grape seeds, and green tea by HPLC/DAD and HPLC/MS

The FO and FC extracts were obtained by an innovative separation process defined as Best Available Technology and recognized by the Environmental Protection Agency [34]. This method consists of an integrated system of several subsequent filtration stages (*i*.*e*. Micro, Ultra, Nano-filtration), followed by reverse osmosis carried out using ecofriendly materials [35]. Oleuropein is the main phenolic compound (14.92% p/p) present in the FO extract. In this study it was obtained from green olive leaves. Oleuropein consists of a secoiridoid core linked to the structure of hydroxytyrosol. The hydrolysis of oleuropein can yield various compounds, such as hydroxytyrosol, known for its important antioxidant activity, deacetoxy oleuropein and elenolic acid, the latter known as a powerful anti-bacterial molecule [36,37]. The polyphenols content in the FO extract is 240.234 mg/g (512.801 μmol/g polyphenols, 14.92% p/p oleuropein) (Table 2).

**Table 2:** Quali-quantitative HPLC/DAD/MS analysis of FO, FC, VN and TV extracts.

**Olive leaves extract**	**mg/g**^**§**^	μ**mol/g**^**§**^	**Grape seeds extract**	**mg/g**	**μmol/g**
hydroxytyrosol glycol	2,352	13,834	gallic acid	0,004	0,024
hydroxytyrosol glucoside	17,373	54,978	catechin dimer B3	2,217	3,836
hydroxytyrosol	2,194	14,245	catechin	11,073	38,183
tyrosol	0,319	2,313	catechin trimer	3,213	3,710
demethyl elenolic acid glucoside	7,067	18,119	catechin dimer B6	2,614	4,522
demethyl elenolic acid diglucoside	13,463	24,390	catechin dimer B2	5,374	9,297
elenolic acid glucoside	4,605	11,399	epicatechin	13,618	46,960
elenolic acid glucoside derivative	2,905	7,191	catechin trimer	3,706	4,280
caffeic acid derivatives	0,475	2,638	epicatechin gallate	6,649	9,108
*p*-cumaroyl acid derivatives	0,422	2,571	epicatechin gallate	6,098	13,796
aesculin	0,483	1,421	catechin tetramers	54,877	47,553
verbascoside	4,726	7,573	epicatechin gallate dimer	180,647	204,816
verbascoside isomer	1,969	3,155	catechin/epicatechin trimers digallate	382,968	327,323
luteolin 7-*O*-glucoside	1,262	2,817	catechin/epicatechin trimers digallate	149,655	127,910
pinoresinol	5,339	14,913	**total polyphenols**	**822,709**	**841,317**
acetoxy pinoresinol	12,131	29,160			
oleuropein	149,158	276,219			
oleuropein derivative	13,993	25,865			
**total polyphenols**	**240,234**	**512,801**	**Artichoke leaves extract**	**mg/g**	**μmol/g**
			monocaffeoyl quinic acids	2,133	6,026
**Green tea leaves extract**	**mg/g**	**μmol/g**	dicaffeoyl quinic acids	4,688	9,085
epigallocatechin gallate	838,840	1831,519	clorogenic acid	16,350	46,188
epicatechin gallate	31,714	71,760	luteolin derivative	2,362	8,260
**total polyphenols**	**870,554**	**1903,279**	**total polyphenols**	**25,534**	**69,559**

^**§**^Polyphenolic composition of olive, green tea, artichoke leaves and grape seeds extracts. The amount of each polyphenol-based molecule is expressed as mg/g and μmol/g.

As for the other extracts, the concentration of total polyphenols expressed as μmol/g was derived by summing the concentrations of each polyphenolic compound, estimated according to their molecular weights and spectrophotometric data. The HPLC/DAD/MS analysis carried out on the FC extract, obtained herein from dried artichoke leaves via a pilot process by hot aqueous extraction, shows the presence of hydroxycinnamic acids (mainly chlorogenic acid and cynarin), and flavones (*e*.*g*. luteolin 7-*O*-glucoside) for a total polyphenols content of 25.534 mg/g (69.559 μmol/g) (Table 2). Moreover, the presence of mono- and di-caffeoyl esters of the quinic acid and flavonoid glycosides was observed in the phenolic fraction, as previously reported [38].

The VN and TV extracts are particularly rich in condensed tannins, which are monomeric or polymeric polyphenolic compounds with widely variable molecular weights based on flavan-3-olic units, such as catechin or epicatechin. Such molecules may be esterified with one or more gallic acid unit/s (*e*.*g*. EGCG). Condensed tannins have a higher stability than hydrolyzable tannins, supporting their multifaceted biological properties [39]. The VN studied here includes a variety of condensed tannins with molecular weights ranging from 290Da (*i*.*e*. catechin and epicatechin) to 1170Da (*i*.*e*. catechin/epicatechin trimers digallate), as well as free gallic acid. In VN extract, the polyphenols amount is 822.702 mg/g (841.317 μmol/g), consisting entirely of condensed tannins (Table 2). The HPLC/DAD and HPLC/MS analysis of the TV extract shows the presence of 870.554 mg/g (1903.279 μmol/g) polyphenols, represented by EGCG and epicatechin gallate (96% and 4% of the total tannins, respectively) (Table 2).

### Polyphenolic extracts inhibit *hrpA* and *psnI* promoter activity *in vitro* without any antibiotic effect

To monitor a potential inhibitory activity of these polyphenolic extracts on the TTSS and QS of *Psn23*, we evaluated their effect on the activation of *hrpA* and *psnI* promoters by using the *gfp*-reporter fusion constructs pLPVM_T3A and pLPVM_QS, respectively. As in other bacteria belonging to the *P*. *syringae* group, the *hrpA* gene encodes the main protein of the TTSS translocating pilus, while *psnI* encodes the *luxI*-homolog lactone synthase of a canonical QS. As reported in Table 3, all the polyphenolic extracts showed, although to a different extent, inhibition of *hrpA* promoter activity when tested at 100 μM.

A reduction of about 48 and 54% was observed for VN and TV, respectively, while a decrease of about 25% was recorded for FO and of 19% for FC. p-coumaric acid (PCA), a plant phenolic compound, was included as positive control at 100 μM as previously reported for the phytopathogenic bacterium *D*. *dadantii* 3937 [40]. We observed that PCA does not cause any significant decrease in *hrpA* promoter activity of *Psn23*, as well as in QS promoter activity (Table 3).

**Table 3:** Effects on bacterial growth, and on the trans-activation of *hrpA* and *psnI* promoters of the polyphenolic extracts and their main constituents.

Extract	Vegetable matrix/main molecule	Bacterial growth^§^	*hrpA* promoter^§^	*psnI* promoter^§^
**VN**	**Grape seeds**	**1.05 ± 0.30**^a^	**0.52 ± 0.11**^ab^	**1.06 ± 0.17**^a^
	Catechin	1.02 ± 0.22^a^	1.08 ± 0.11^a^	0.96 ± 0.27^a^
	Epicathechin	0.99 ± 0.19^a^	1.12 ± 0.13^a^	1.07 ± 0.32^a^
**TV**	**Green tea leaves**	**1.01 ± 0.16**^a^	**0.46 ± 0.13**^b^	**0.79 ± 0.14**^ab^
	Epigallocatechin gallate	1.00 ± 0.17^a^	0.59 ± 0.24^ab^	0.94 ± 0.12^a^
**FO**	**Olive leaves**	**1.33 ± 0.16**^a^	**0.75 ± 0.18**^a^	**0.68 ± 0.16**^ab^
	Oleuropein	1.00 ± 0.15^a^	1.24 ± 0.18^a^	1.01 ± 0.25^a^
	Hydroxytyrosol	0.98 ± 0.18^a^	0.49 ± 0.17^b^	0.63 ± 0.16^ab^
	Luteolin 7-*O*-glucoside	1.32 ± 0.12^a^	1.18 ± 0.10^a^	0.92 ± 0.18^a^
**FC**	**Artichoke leaves**	**1.45 ± 0.18**^a^	**0.81 ± 0.17**^a^	**0.57 ± 0.13**^ab^
	Caffeic acid	1.16 ± 0.13^a^	0.99 ± 0.10^a^	0.94 ± 0.17^a^
	Chlorogenic acid	1.13 ± 0.15^a^	1.02 ± 0.12^a^	1.08 ± 0.20^a^
	Cynarine	1.22 ± 0.10^a^	1.10 ± 0.32^a^	1.03 ± 0.29^a^
	**Kanamycin**^**˄**^	**0.46 ± 0.18**^b^	**0.21 ± 0.18**^b^	**0.23 ± 0.22**^b^
	**p-Coumaric acid**^**˄**^	**0.92 ± 0.15**^a^	**1.03 ± 0.22**^a^	**0.98 ± 0.25**^a^

^§^OD_600_ was recorded after 24h growth and data are calculated as GFP Abs _(Ex.485nm; Em.535nm)_ / Abs _(600nm)_ ± SD, and as normalized fold versus untreated bacterial cultures.

^˄^ Kanamycin and p-coumaric acid (PCA) were used as negative and positive control, respectively.

Common letters in correspondence of each chemical compound indicate differences not statistically significant at *P* <0.05 according to Tukey’s test.

Furthermore, we found that FC, FO and TV inhibit *psnI* promoter’s activity at a different extent (about 43%, 32%, and 21%, respectively), while a slight increase is obtained in the presence of VN. No negative effect on bacterial growth was observed with VN and TV, and a growth increase was recorded after treatment with FO and FC (33 and 45%, respectively). These data indicate that the inhibitory effect of these polyphenolic plant extracts on TTSS and QS may not be a consequence of their negative impact on bacterial growth. Conversely, the antibiotic kanamycin used as negative control causes both inhibition on bacterial growth, and on TTSS and QS promoter activities (Table 3). Overall, all the extracts tested had a higher inhibitory activity on TTSS rather than on QS. In particular, the extracts VN and TV are the most effective in reducing the *in vitro* activation of the *hrpA* promoter.

For comparison, the inhibitory activity against TTSS and QS of the corresponding bioactive molecules for each of the polyphenolic extracts here examined (*i*.*e*. catechin, epicatechin, EGCG, oleuropein, caffeic acid, chlorogenic acid, cynarine, luteolin 7-O glucoside) was also analyzed. None of these molecules affect bacterial growth, and all but hydroxytyrosol and EGCG (Table 3) show a significant inhibitory effect on *hrpA* and *psnI* promoters. A reduction of *hrpA* and *psnI* promoter activity was found with hydroxytyrosol (51 and 37%, respectively), and with EGCG (41 and 6%, respectively), which represent a considerable fraction of TV and FO extracts. However, both EGCG and hydroxytyrosol had a higher concentration when tested individually than that in TV and FO extracts, respectively.

### Polyphenolic extracts inhibit HR in tobacco and knot development in oleander

To exclude any phytotoxic effect of the polyphenolic extracts here used, they were infiltrated into the mesophyll of tobacco leaves, without observing any unspecific phytotoxicity (data not shown).

Moreover, when these extracts have been co-infiltrated at 100 μM with *Psn23* wild type cells, a strong reduction in HR symptoms was found when in presence of TV or VN in comparison to the infiltration with *Psn23* alone (S1 Fig), and comparable to the results of the non pathogenic ∆*hrpA* mutant [24]. Similarly a reduced HR was obtained in presence of FC or FO, although to a lesser extent than with TV or VN (S1 Fig). Therefore, these data further confirm the inhibiting activity of the polyphenolic extracts on the *Psn23* TTSS *in vitro* and *in planta*, where HR elicitation by *Psn23* was suppressed.

To determine whether these polyphenolic extracts could prevent the development of knot disease symptoms in oleander plants, an *in vitro* plant model system was developed using explants from 2-year old twigs of *N*. *oleander*.

**Fig 1:**
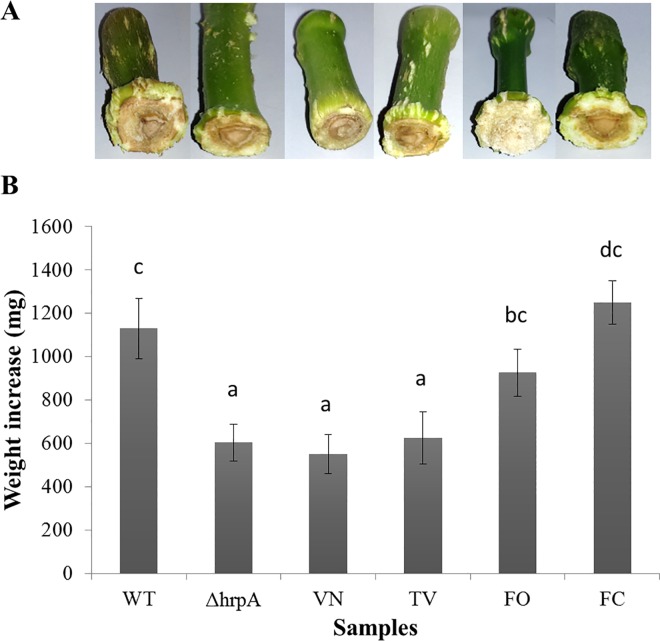
Pathogenicity test with *Psn23* on oleander explants, following treatment with polyphenolic extracts VN, TV, FO or FC. Explants from adult oleander plants were inoculated with *P*. *savastanoi* pv. *nerii* strain *Psn23*, in the presence or absence of the VN, TV, FO or FC extracts (100 μM). As negative control the non pathogenic mutant ∆*hrpA* was used. (A) Development of hyperplastic knots at 21 dpi with (from left to right): *Psn23*, ∆*hrpA*, *Psn23+*VN, *Psn23+*TV, *Psn23+*FO, *Psn23+*FC. The symptoms are detectable as swelling at the inoculated end of oleander explants. (B) Normalized weight increase of oleander explants at 21 dpi inoculated with (from left to right): *Psn23*, ∆*hrpA*, *Psn23+*VN, *Psn23+*TV, *Psn23+*FO, *Psn23+*FC. Values are means ± SD of nine replicates for each treatment. Different letters indicate significant differences among means at *P* < 0.05, according to Tukey's test.

As shown in Fig 1A, a significant difference in the symptoms development was observed following inoculation of *Psn23* wild type bacteria with VN or TV. In particular, a reduction of more than half of the explant weight increase was obtained for oleander explants inoculated with *Psn23* treated with VN or TV in comparison to those untreated (Fig 1B).

These data were confirmed by monitoring the *in planta* bacterial growth rate at 21 dpi, where a strong decrease of bacterial multiplication was recorded following inoculation of *Psn23* with VN or TV extracts, comparable to the *in planta* growth of ∆*hrpA* mutant (S3 Fig).

### Polyphenolic extracts alter TTSS and QS gene expression

**Fig 2:**
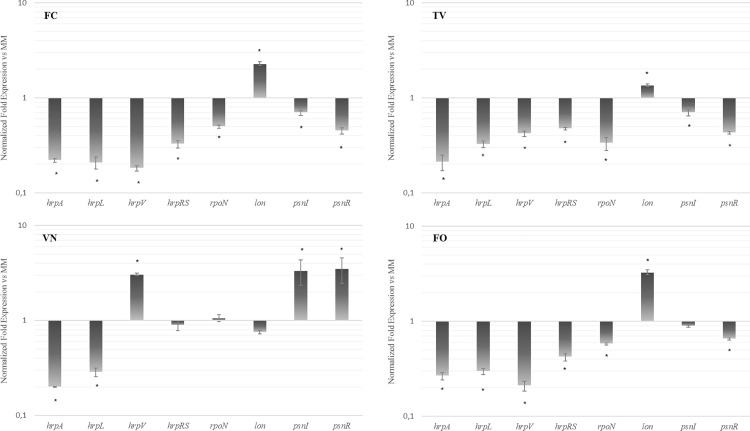
Relative gene expression analysis of key genes correlated to TTSS and QS of *Psn23*. Relative mRNA levels of *hrpA*, *hrpL*, *hrpV*, *hrpRS*, *rpoN*, *lon*, *psnI*, *psnR* genes of *Psn23*, grown in MM supplemented with the polyphenolic extracts TV, VN, FO or FC compared to levels in MM alone (untreated). The data are expressed as the average of three replicates ± SD. Asterisks indicate significant differences compared with the untreated sample at *P* <0.05.

To determine the effect of these polyphenolic extracts on the modulation of TTSS and QS pathways of *P*. *savastanoi* pv. *nerii*, we performed a gene expression analysis by real-time PCR. The whole sequence and organization of the TTSS cluster of *Psn23* has been previously reported [29], and the QS genomic organization is available as well (GenBank Accession Number FR717654). To induce *in vitro* the expression of genes related to the TTSS, *Psn23* was grown on MM, which is considered to mimic plant apoplastic conditions [41]. This medium was supplemented with 100 μM of VN, TV, FO or FC. The expression of the gene *hrpA*, coding for the main component of TTSS pilus, was evaluated as well as that of several genes known to be involved in TTSS regulation, such as *hrpL*, *hrpV*, *hrpRS*, *rpoN* and *lon* [42]. As shown in Fig 2, all the polyphenolic extracts here examined strongly reduce *hrpA* mRNA levels.

Besides its role in the assembly of the TTSS pilus, HrpA was hypothesised to regulate *hrpRS* gene transcription, in a way that still remains to be determined, presumably through a positive feedback on *hrpRS* [43]. Consistently with these data, *hrpRS* expression is also negatively affected as well as the *hrpL* mRNA levels (Fig 2). However, it is likely that TV, FO and FC inhibit TTSS expression in the same fashion, while VN impairs TTSS functionality otherwise. The characteristic effects of these polyphenolic extracts on the proper TTSS activation may be attributed to their different impact on the two main regulatory mechanisms that finely control the expression of *hrp* cluster in *P*. *syringae*, *i*.*e*. HrpRS and GacS/GacA system [44]. The HrpS/HrpR heterodimer is crucial for the transcriptional activation of *hrpL*, which is also under the positive control of RpoN [45]. The *rpoN* transcription as well as that of *hrpRS* are activated by the GacS/GacA system, although the mechanism through which GacA regulates the expression of *hrpRS* and *rpoN* are still unknown [44]. Concerning negative regulators, HrpV controls *hrp* cascade upstream to HrpRS through a protein-protein interaction between HrpV and HrpS [46], while HrpR is specifically degraded by the Lon protease, and both of them depend on the HrpRS cascade. As shown in Fig 2, we observed a significantly lower amount of *hrpL*, *hrpV* and *hrpRS* mRNA levels in *Psn23* grown in MM supplemented with TV, FO and FC, in comparison to levels found when *Psn23* was grown in MM alone. Moreover, TV, FO and FC promote the decrease of *rpoN* mRNA levels, while the *lon* mRNA levels are almost unaffected, suggesting the involvement of GacA-RpoN-HrpL pathway [47,48]. In contrast, VN appears to compromise *hrp* cascade through the GacA-HrpRS-HrpL pathway, as suggested by the increase of *hrpV* mRNA level, while *rpoN* expression in *Psn23* grown in MM supplemented with VN is not statistically different to the expression level observed in MM alone (Fig 2). Lastly, we investigated the effect of these polyphenolic extracts on the two genes *psnI* and *psnR*, both correlated with QS. In Gram-negative bacteria, the first encodes for an acyl homoserine lactone synthase that belongs in most cases to the LuxI-protein family and produces the most common signal molecule, *i*.*e*. N-acyl homoserine lactone (AHL). The second encodes for a transcriptional sensor/regulator belonging to the LuxR family that forms a complex with the cognate AHL at threshold (quorum) concentrations, thereby affecting the transcription of target genes [49]. As shown in Fig 2, the data obtained corroborate those previously reported on *psnI* promoter activity. Namely, TV and FC were demonstrated to statistically reduce the transcript levels of both *psnR* and *psnI*, while FO only *psnR*. Conversely, VN strongly enhances both *psnR* and *psnI* expression. Moreover, the data further confirm the involvement of the GacA-HrpRS-HrpL pathway as a putative target of VN, and are in agreement with the tight functional link between TTSS and QS regulation mediated by GacA/GacS as already reported for *P*. *syringae* pv. *tomato* DC3000 [48].

To the best of our knowledge, this is the first wide gene expression study in which the effects of polyphenolic extracts from grape seeds, green tea, olive, and artichoke have been investigated against a wide set of genes correlated to TTSS and QS in *P*. *savastanoi* pv. *nerii*.

### Polyphenolic extracts inhibit Type Three Secretion System pilus assembly

To further demonstrate the highly specific effect of the VN, TV, FO or FC polyphenolic extracts on TTSS machinery, we investigated their impact on the TTSS pilus assembly. To this purpose, we set up and performed a Congo red-based assay on *Psn23*-treated cultures, to quantitatively evaluate any variation in the presence of different types of pili and fimbriae, including the TTSS pilus [50]. Congo red binding has been shown to be associated with the presence of different types of bacterial appendages, although the basis for this phenomenon is unclear [51]. Furthermore, Congo red staining has been demonstrated to be a fast and economical method for monitoring TTSS assembly also in *P*. *syringae* pv. *tomato* [31].

**Fig 3:**
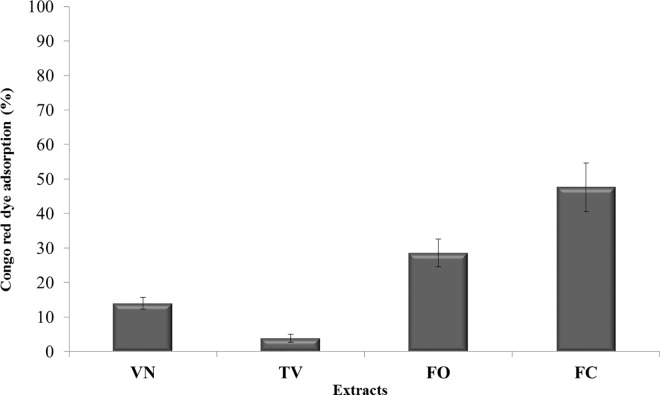
Effect of polyphenolic extracts on Congo red dye absorption of *Psn23* bacterial cultures. Percentage of Congo red dye absorption of *Psn23* bacterial cultures, grown in MM amended with the polyphenolic extracts VN, TV, FO or FC. The data were calculated according to the formula: [(X_unk_−X_ΔhrpA_) / (X_WT_−X_ΔhrpA_)] *100 where X_WT_ and X_ΔhrpA_ are the ratio OD_490_/OD_600_ for *Psn23* and *ΔhrpA* respectively. The data represent the means ± SD of three replicates. All treatments are statistically significant (*P* <0.05).

The data obtained show that VN and TV cause a reduction of the dye absorption by *Psn23*-treated cells, corresponding to 86% and 96%, respectively (Fig 3). In the case of FO and FC, the Congo red absorption was reduced to about 71% and 52%, respectively (Fig 3). Overall, such a decrease in Congo red binding to *Psn23*-treated cells in comparison to those untreated, indirectly demonstrates that these polyphenolic extracts, although with different effectiveness, compromise the correct assembly of the TTSS pilus.

**Fig 4:**
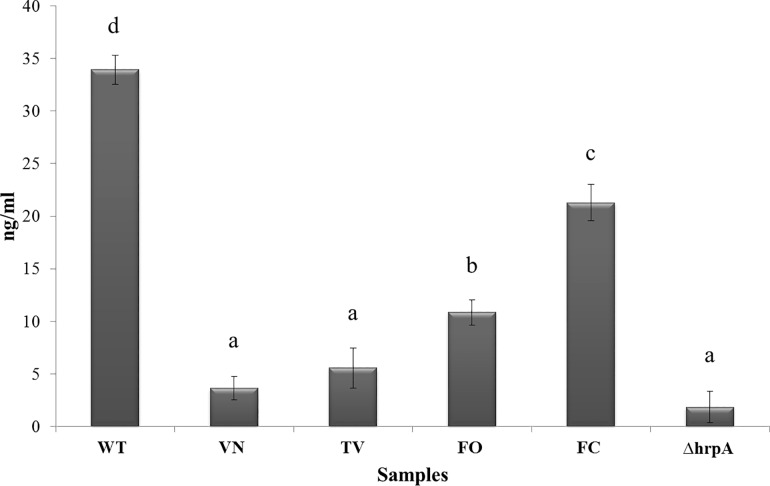
ELISA assay on *Psn23* bacterial supernatant amended with polyphenolic extracts. Quantification of HrpA protein by ELISA assay on bacterial supernatant of wild type *Psn23* grown on MM, or on MM amended with the polyphenolic extracts VN, TV, FO, or FC. As a negative control the ∆*hrpA* mutant was used. As a reference for quantification, a standard curve was established by a serial dilution of the *Psn23* HrpA recombinant protein (117 pg/ml– 40 ng/ml). The data represent the means ± SD of three replicates. Statistically significant differences are represented by different letters above the bars (ANOVA and Tukey’s test, *P* < 0.05).

To confirm and directly verify these findings, the amount of HrpA produced by *Psn23* in the supernatant was also quantified by ELISA, following or not treatment with the VN, TV, FO or FC extracts. The results obtained are consistent with those from Congo red assay. In particular, in the supernatants of untreated *Psn23* cells a concentration of 33.93 ng/ml of HrpA protein was detected (Fig 4). In contrast, when TV or VN were supplemented to MM, the HrpA concentrations in the supernatants were 5.58 and 3.68 ng/ml, respectively, thus comparable to the levels detected for the ∆*hrpA* mutant (1.85 ng/ml). Following the treatment with FO or FC, HrpA concentrations of 10.87 and 21.30 ng/ml, respectively, were found (Fig 4). In conclusion, these findings demonstrate that the polyphenolic extracts obtained and tested in our work are able to interfere in a very specific manner with TTSS, as previously indicated by the results on gene expression analysis of this master pathogenicity system.

### Evaluation of toxic effects of polyphenolic compounds at the molecular level

In view of the potential application of these polyphenolic extracts in plant disease control, we evaluated the toxicity of several polyphenols used here as reference *i*.*e*. EGCG, catechin, oleuropein, hydroxytyrosol and chlorogenic acid. In particular, these compounds were examined for their effects on the transport activity of SR Ca^2+^-ATPase, which is a crucial molecular target in a variety of physiological processes. SR Ca^2+-^ATPase belongs to the highly-conserved P-type ATPase family. P-type ATPases are a large, ubiquitous and varied family of membrane proteins that are involved in many transport processes in virtually all living organisms [52]. SR Ca^2+-^ATPase couples the hydrolysis of one molecule of ATP to the active transport of two Ca^2+^ ions from the cytoplasm to the lumen of SR. The Ca^2+-^ATPase transport activity plays a major role in cell Ca^2+^ signaling and homeostasis in both eukaryotes and prokaryotes [53,54]. In our study we employed SSM-based electrophysiology to compare the effects of the polyphenolic compounds with the inhibitory action of copper ions (Cu^2+^) towards the SR Ca^2+^-ATPase. In fact, several heavy metal ions, including Cu^2+^, were found to inhibit Ca^2+^-ATPase activity in different types of membranes [55]. Such inhibition typically causes a sudden increase in the cytosolic concentration of calcium ions, endoplasmic reticulum stress, and eventual cell death through apoptosis.

**Fig 5:**
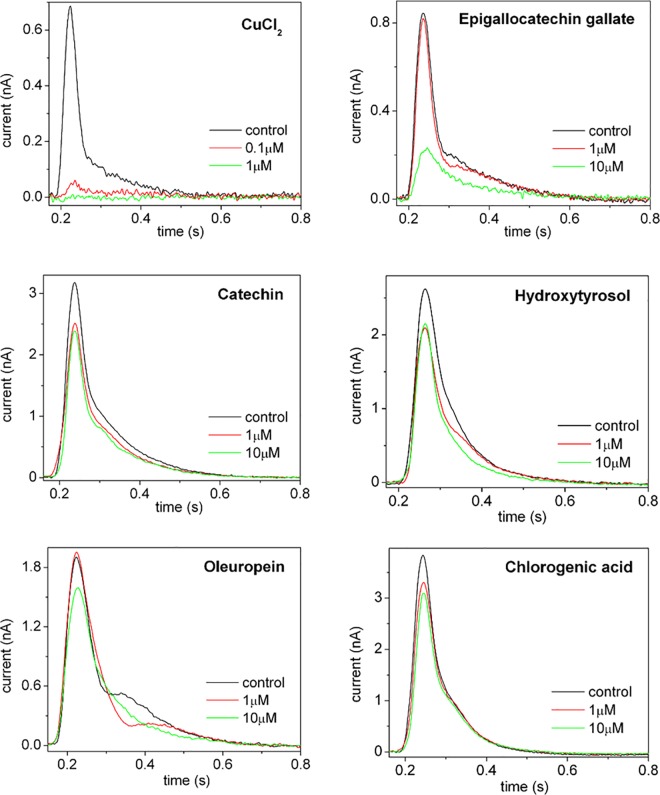
Current measurements on SR vesicles adsorbed on a SSM. Current signals induced by 100 μM ATP concentration jumps in the presence of 10 μM Ca^2+^_free_ and in the absence (black curve, control measurement) or in the presence of CuCl_2_ (red curve) or of the polyphenolic compounds EGCG, catechin, oleuropein, hydroxytyrosol and chlorogenic acid (green curves).

To investigate the interaction of these polyphenolic compounds with SR Ca^2+^-ATPase and its possible inhibition, we performed current measurements on SR vesicles adsorbed on a SSM. The SSM technique allows direct measurements of charge displacements within the transport protein yielding valuable information about the ion transport mechanism [32,56,57]. The technique is also well suited for the analysis of inhibitor interactions with membrane transporters [33,58]. As shown in Fig 5, a current signal was observed following a 100 μM ATP concentration jump in the presence of CaCl_2_ (10 μM), taken as a control measurement. It is worth mentioning that the charge obtained by numerical integration of the ATP-induced current signal is attributed to an electrogenic event corresponding to translocation and release of bound Ca^2+^, after utilization of ATP [32,33,57].

ATP concentration jump experiments were then performed in the presence of CaCl_2_ and copper (Cu^2+^) or the polyphenolic compounds at different concentrations. The corresponding ATP-induced current signals were then compared to the control measurement obtained in the absence of these substances. In the case of CuCl_2_, we found that at 0.1 μM concentration Cu^2+^ ions suppress almost completely the ATP-induced current signal and the related displaced charge (Fig 5). Therefore, we may conclude that sub-micromolar copper exerts a remarkable inhibitory effect on SR Ca^2+-^ATPase by interfering with ATP-dependent calcium translocation through the enzyme.

On the other hand, the polyphenol-based molecules here studied have minor, if any, effects on the ATP-induced current signal over a concentration range from 1 to 10 μM, with the exception of EGCG (Fig 5). In fact, in the case of EGCG a significant reduction of the current amplitude was recorded at 10 μM EGCG. Such an interference with ATP-dependent Ca^2+^ translocation in the presence of a high EGCG concentration has been reported in recent biochemical studies [59,60]. In particular, EGCG was found to inhibit both Ca^2+^ uptake rate and ATPase activity with half-maximal effects observed at ~12 μM [60] and ~16 μM [59]. In these studies, however, no inhibitory effect of EGCG on SR Ca^2+-^ATPase activity was reported in the concentration range between 0.1 and 1 μM.

Therefore, our results indicate that as compared to copper, the polyphenolic compounds here investigated do not affect the SR Ca^2+-^ATPase transport activity in the sub-micromolar concentration range.

## Conclusions

The identification and development of new ecofriendly alternatives for plant protection is becoming increasingly important, especially to reduce the use of copper compounds which are widely employed in agriculture practice, as well as to limit the emergence of copper-resistant strains. Every year the agricultural industry generates billions of metric tons of plant biomass and waste, which can be environmentally polluting if not properly managed.

Currently, several systems for kilo-scale extraction and fractionation of natural active ingredients from plant by-products were proposed [61]. The optimization of industrial closed cycle platforms for the recovery of green chemicals has been so far of interest for innovative applications in feed, food, as well as for cosmetic and nutraceutical industry. The results reported here demonstrated their potential and effective use in plant protection as well, which may lead to the development of sustainable models of circular economy into the agricultural sector.

In this study, we have demonstrated that standardized polyphenolic extracts from *O*. *europaea*, *C*. *scolymus* leaves, *V*. *vinifera* seeds and *C*. *sinensis* leaves, characterized by HPLC/DAD and HPLC/MS analysis, are able to inhibit specifically the TTSS and partially the QS of *P*. *savastanoi* strain *Psn23*. A close relationship was found among the data obtained through promoter activation and gene expression analysis, both for TTSS and QS. The *in vitro* anti-microbial activity of olive mill wastewater on the growth of *P*. *savastanoi* pv. *savastanoi* was already known [62], but in this study for the first time several polyphenolic extracts were successfully examined for their anti-virulence activity against this plant pathogenic bacterium. The additive and synergistic effects of polyphenolic extracts are responsible for their powerful bioactive properties and thus their effectiveness has to be attributed to the complex mixture of phytochemicals present in whole extract. Furthermore, we have shown that these extracts compromise the TTSS pilus assembly in a very specific manner without undermining bacterial viability. Finally, the absence of any significant toxicity on SR Ca^2+-^ATPase supports the potential of this innovative strategy, which aims at employing standardized natural polyphenolic extracts as effective copper substitutes in the control and management of bacterial diseases of plants.

## Supporting Information

S1 Fig Effect of polyphenolic extracts on HR development on tobacco leaves. Hypersensitive Response assay on tobacco leaves at 48-h after co-infiltration of *Psn23* wild type bacteria (yellow rings), with FO, FC, VN or TV polyphenolic extracts (white rings). As control, sterile physiological solution was used (black ring).
(TIF)

S2 Fig qPCR melting curves for each primer pairs tested. Blue scaling color lines correspond to serial dilutions of target gene, red lines correspond to negative control (DNA-free sterile distilled water).
(TIF)

S3 Fig *In planta* bacterial growth rate of *Psn23* treated with polyphenolic extracts VN, TV, FO or FC. Bacterial multiplication was monitored at 21 dpi. Values are means ± SD of nine replicates for each treatment. Different letters indicate significant differences among means at *P* < 0.05, according to Tukey's test.
(TIF)

S1 Table Primers used in this study for recombinant plasmids construction. (PDF)

S2 Table Sequences, temperature of melting (Tm°), amplicon size (bp), efficiency, R^2^, slope and Ct values of the primers used in real-time PCR. (PDF)
