# Supplementary figures and images for: Global Analysis of Type Three Secretion System and Quorum Sensing Inhibition of Pseudomonas savastanoi by Polyphenols Extracts from Vegetable Residues
Source: PLoS One. 2016 Sep 26;11(9):e0163357. doi: 10.1371/journal.pone.0163357 (PMC5036890; doi:10.1371/journal.pone.0163357)

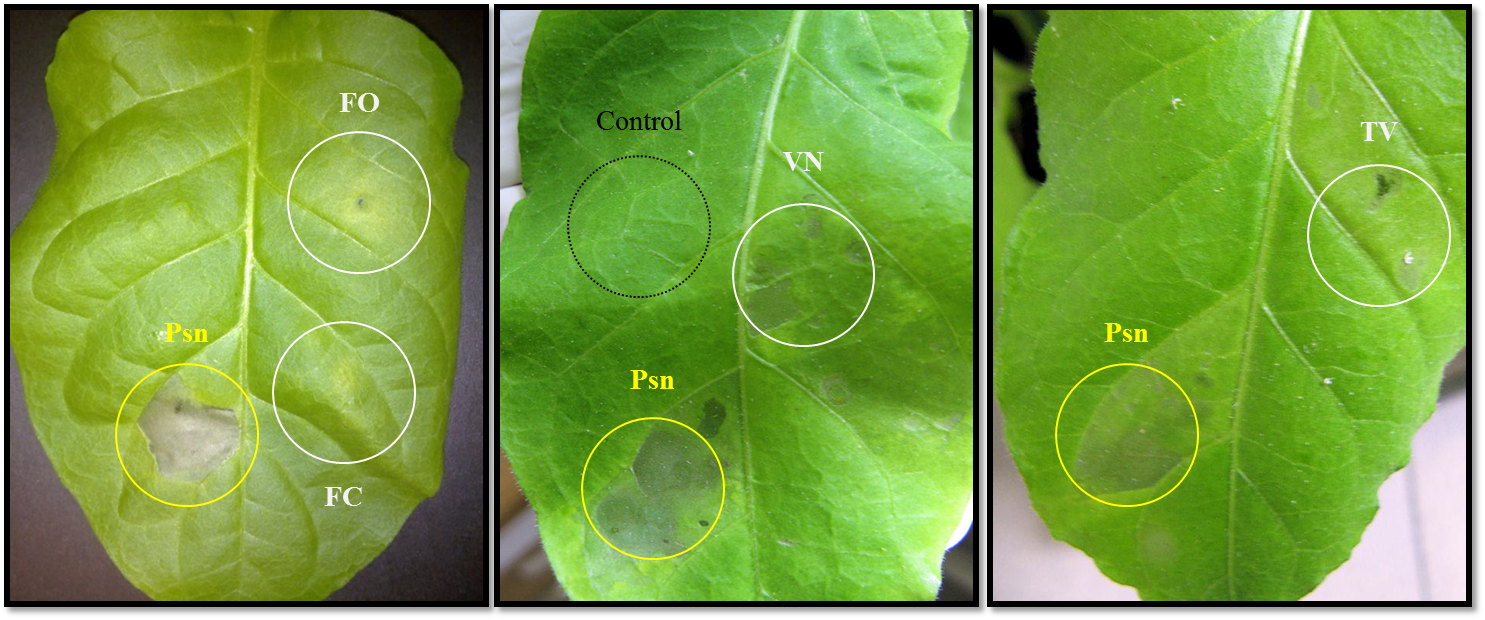

Supplement: S1 Fig — Hypersensitive Response assay on tobacco leaves at 48-h after co-infiltration of Psn23 wild type bacteria (yellow rings), with FO, FC, VN or TV polyphenolic extracts (white rings). As control, sterile physiological solution was used (black ring). (TIF) [file pone.0163357.s001.tif]

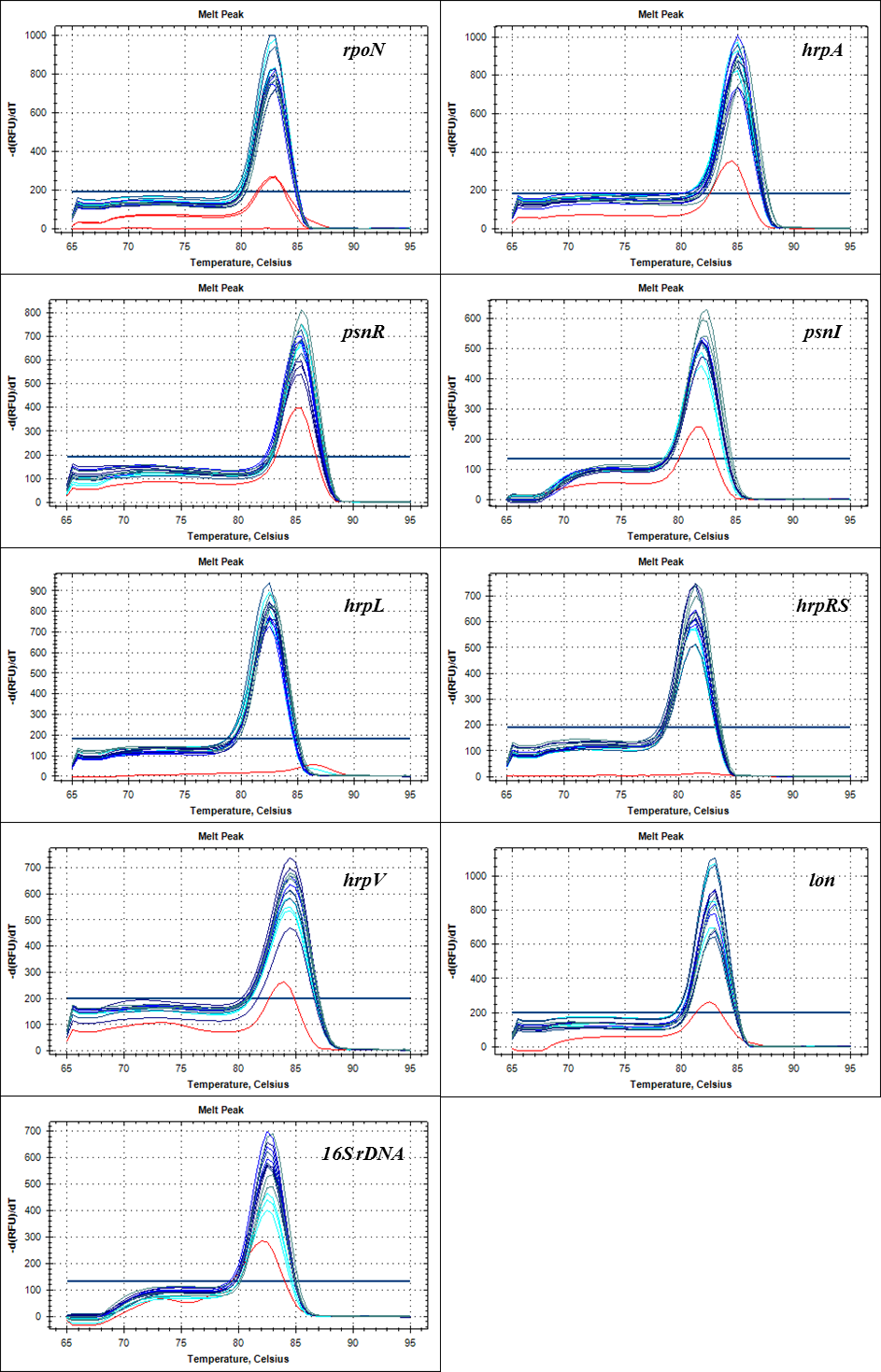

Supplement: S2 Fig — Blue scaling color lines correspond to serial dilutions of target gene, red lines correspond to negative control (DNA-free sterile distilled water). (TIF) [file pone.0163357.s002.tif]

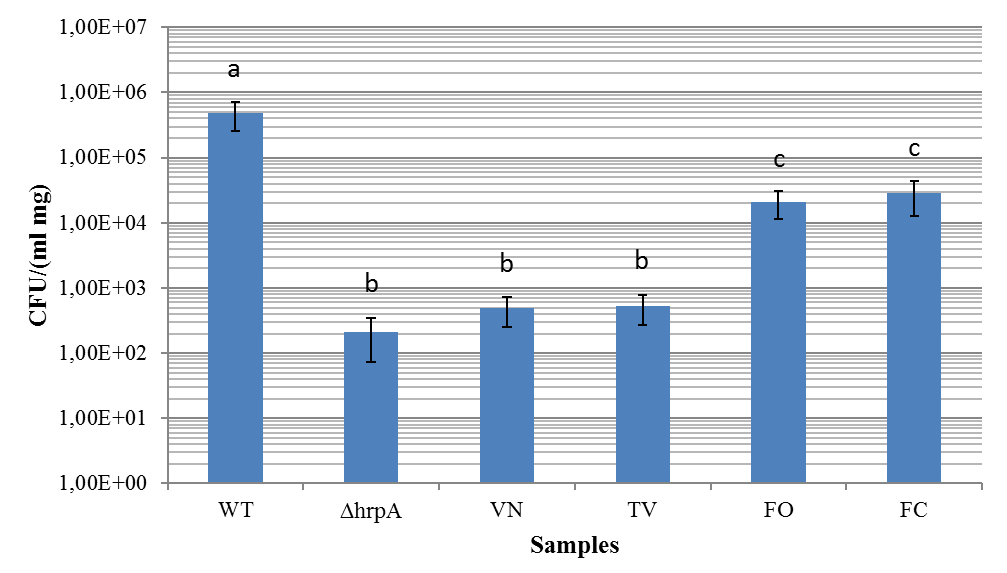

Supplement: S3 Fig — Bacterial multiplication was monitored at 21 dpi. Values are means ± SD of nine replicates for each treatment. Different letters indicate significant differences among means at P < 0.05, according to Tukey's test. (TIF) [file pone.0163357.s003.tif]
