# Supplementary material for: Global Analysis of Type Three Secretion System and Quorum Sensing Inhibition of Pseudomonas savastanoi by Polyphenols Extracts from Vegetable Residues
Source: PLoS One. 2016 Sep 26;11(9):e0163357. doi: 10.1371/journal.pone.0163357 (PMC5036890; doi:10.1371/journal.pone.0163357)
Supplement: S1 Table — (PDF) [file pone.0163357.s004.pdf]

Primer name	Primer sequence (5’→3’)	Tm°
GFP_BamHI_For	AAAGGATCCATGGTGAGCAAGGGCG	62.1
GFP_KpnI_Rev	AAAGGTACCTTACTTGTACAGCTCGTC	60.2
T3_XbaI_For	AAATCTAGATTTTTTGCAGAGCGCT	62.6
T3_BamHI_Rev	AAAGGATCCCTAAATTCAAACAACGTG	64.0
QS_XbaI_For	AAATCTAGACGACATAGGCACTTCC	60.0
QS_BamHI_Rev	AAAGGATCCTATAAACTCCACTTCGCA	62.8
