# Supplementary material for: Global Analysis of Type Three Secretion System and Quorum Sensing Inhibition of Pseudomonas savastanoi by Polyphenols Extracts from Vegetable Residues
Source: PLoS One. 2016 Sep 26;11(9):e0163357. doi: 10.1371/journal.pone.0163357 (PMC5036890; doi:10.1371/journal.pone.0163357)
Supplement: S2 Table — (PDF) [file pone.0163357.s005.pdf]

Gene	Primer name	Tm °C	Primer sequence (5'→3') (bp)	Amplicon size (bp)	Efficiency (%)	R^2	Slope	Ct minor	Ct major
hrpA	hrpA_RT_for	62.7	GCAGGGTATCAACAGCGTCAAG	156	102.6	0.998	- 3.258	23.85	34.75
	hrpA_RT_rev	63.0	CCGTTCTCTTCGTTCGCAGTG						
hrpL	hrpL_RT_for	59.7	GTATTGCGTTGAACCTGAT	126	104.6	0.994	- 3.216	25.83	32.56
	hrpL_RT_rev	59.7	CGTCTACCTGATGAGTGATA						
hrpV	hrpV_RT_for	61.3	GAGCGGTTCCGTAACTAC	130	105.2	0.990	- 3.211	26.50	33.26
	hrpV_RT_rev	61.6	CTGCCAGCATCAACTCAT						
hrpRS	hrpRS_RT_for	61.2	ACCCGCAGAGTGAAGAAC	88	99.8	0.998	- 3.265	23.72	31.11
	hrpRS_RT_rev	62.0	CGCTTGAGTGACTGTTGAATC						
rpoN	rpoN_RT_for	60.0	CTACCGTGGATAACCTTGA	125	104.2	0.991	- 3.219	25.73	32.16
	rpoN_RT_rev	60.3	GTCATCATCGTTGCTTGG						
lon	lon_RT_for	61.3	CCGAGCAGAACCATAACTT	134	103.2	0.995	- 3.198	25.52	34.21
	lon_RT_rev	61.2	CAGGCGAATGACTTCCAT						
psnI	pssI_RT_for	61.0	ACGGTGGTCAGCAAGGCAATG	161	102.1	0.996	- 3.287	23.49	35.15
	pssI_RT_rev	61.0	GCCAACGGAGCAGGTCATCC						
psnR	pssR_RT_for	61.3	AATGGCGTAATGCTATGC	162	103.9	0.991	- 3.232	25.24	32.43
	pssR_RT_rev	60.7	TGGCGATTTCACTTATGC						
16s rDNA	16s_RT_for	63.7	GGAATCTCGCTGGTAGTGGGG	157	103.7	0.998	- 3.289	19.31	33.49
	16s_RT_rev	64.0	ATCGTCGCCTTGGTGAGCC						
